# Blood group phenotyping and associated sepsis across childhood stages: A case-control study

**DOI:** 10.1097/MD.0000000000046713

**Published:** 2025-12-19

**Authors:** Howaida M. Hagag, Maha M. Bakhuraysah, Mustafa A. Alswat, Abdulrahman A. Almalki, Ahmad M. Homsi, Narjes A. Alhassan, Shmokh K. Altalhi, Nada A. Albogami, Amjad S. Alrubaie, Maram Jameel Hulbah, Reem Amr Ramadan, Khadiga A. Ismail, Usama Mahmoud Marzouk, Abdalla A. Elnour, Mahmoud Yehyia Sleem, Raed A. Alharbi, Ali A. Lafi Alghamdi, Salem A. Turki Alghamdi, Abdulkarim Hasan

**Affiliations:** a Clinical Laboratory Sciences Department, College of Applied Medical Sciences, Taif University, Taif, Saudi Arabia; b Laboratory and Blood Bank Department, Children Hospital, Taif Health Cluster, Taif, Saudi Arabia; c Internal Medicine, Faculty of Medicine, Ain-Shams University, Cairo, Egypt; d Department of Clinical Biochemistry, Faculty of Medicine, King Abdulaziz University, Rabigh, Saudi Arabia; e Hematology Department, Regional Laboratory and Central Blood Bank, Ministry of Health, Al-Madinah Al-Munawarah, Saudi Arabia; f Department of Laboratory Medicine, Faculty of Applied Medical Sciences, Al-Baha University, Albaha, Saudi Arabia; g Laboratory Department, Prince Mishari bin Saud Hospital in Baljurashi, Al-Baha Health Cluster, Ministry of Health, Al-Baha, Saudi Arabia; h Pathology Department, Faculty of Medicine, Al-Azhar University, Cairo, Egypt.

**Keywords:** antimicrobial susceptibility, bacteria sepsis, blood groups, Taif

## Abstract

Variations in blood group antigens may influence susceptibility to infections, as certain antigens can act as microbial receptors. Sepsis remains a major cause of morbidity and mortality in pediatric populations; however, the relationship between blood group phenotypes and infection risk is not fully elucidated. This study aimed to investigate the association between blood group phenotypes and sepsis across different pediatric age groups. A case-control study was conducted involving 302 pediatric patients, including 60 with sepsis (cases) and 242 with non-septic illnesses (controls). Blood group phenotyping (ABO, Rh, Kell), complete blood count, blood cultures, and antimicrobial susceptibility testing for culture-positive samples were performed. The mean age of participants was 2.59 ± 2.07 years, comprising 22 newborns (7%), 118 infants (39%), and 162 children (54%). Among sepsis cases, the mean age was 1.78 ± 1.58 years, with 48.3% being male. In the control group, the mean age was 2.15 ± 2.78 years, and 59.5% were male. Blood group O was the most prevalent (55.3%), followed by A (24.8%), B (15.6%), and AB (4.3%). The most frequently isolated pathogens were Gram-positive cocci (45%), followed by Gram-negative bacilli (31.6%), *Candida* species (13.3%), and mixed infections (10%). A statistically significant association was observed between the AB blood group and susceptibility to specific microorganisms, particularly Gram-negative bacilli and multidrug-resistant (MDR) strains. This study highlights a significant association between the AB blood group and increased susceptibility to certain pathogens in pediatric sepsis. These findings warrant further large-scale investigations to better define the role of blood group phenotypes in infection risk among children.

## 1. Introduction

The immune system undergoes dynamic development throughout the fetal period and early childhood. During gestation and around birth, the fetal immune environment is characterized by a tolerogenic state and a predominance of T helper 2 (Th2) cell polarization. Maternal immunoglobulin G (IgG) antibodies are transferred transplacentally and provide passive immunity to the neonate during the first months of life. At birth, neonatal B cells and innate immune cells display phenotypic characteristics similar to those of adults. Postnatally, especially after weaning, the immune system continues to mature, marked by an increase in regulatory T cells (Tregs) and a gradual shift toward a balanced Th1/Th2 response.^[1]^

Throughout infancy, the immune system develops progressively. Maternal IgG antibodies – transferred both transplacentally and through breastfeeding – play a crucial role in early protection against pathogens previously encountered by the mother. As maternal antibody levels wane, infants become more vulnerable to infections. However, this period also coincides with the maturation of the innate and adaptive immune responses. Vaccination during early childhood is vital for stimulating the development of long-term adaptive immunity and reducing the incidence of vaccine-preventable infectious diseases.^[2]^

Sepsis is defined as the presence of systemic inflammatory response syndrome secondary to suspected or proven infection. Blood samples for culture were collected within 1 hour of sepsis suspicion and prior to antibiotic administration. It remains a leading cause of morbidity and mortality in children globally. It is particularly concerning in pediatric populations, with a reported prevalence of 8.2% and a mortality rate approaching 25% in some settings. Due to the variable microbiological patterns of sepsis, research into causative pathogens and their antimicrobial susceptibility profiles is essential to guide effective treatment strategies.^[3,4]^

Blood group antigens, beyond their role in transfusion medicine, can act as microbial receptors or co-factors, influencing susceptibility to infection. The interaction between specific pathogens and blood group antigens underscores the complex role of host genetic factors in disease pathogenesis and immune evasion. Understanding these associations could support the development of targeted prevention and treatment approaches for infectious diseases.^[4,5]^

A study examining the relationship between ABO blood groups and susceptibility to nosocomial infections found that individuals with blood groups B and AB were more prone to Escherichia coli infections, while those with blood group A had higher susceptibility to Staphylococcus aureus and Pseudomonas aeruginosa. In particular, individuals with blood group AB appeared especially vulnerable to P. aeruginosa infection.^[6]^

Some bacterial pathogens, such as E. coli O125, Morganella morganii, Enterococcus faecalis, and Mycobacterium tuberculosis, have been reported to trigger immune responses that cross-react with the Kell antigen. This cross-reactivity is hypothesized to result from bacterial proteins structurally similar to the human enzyme neprilysin, providing a plausible mechanism for the observed immunological reaction.^[7]^

Additionally, Pseudomonas aeruginosa expresses lectins (e.g., PA-IL and PA-IIL) that bind galactose- and fucose/mannose-containing glycoconjugates, facilitating host colonization. Several other pathogens – including Helicobacter pylori, Haemophilus influenzae, Neisseria meningitidis, and Streptococcus pneumoniae – have also been shown to interact with ABO antigens, further supporting the connection between blood group phenotype and infection susceptibility.^[8]^

Ahmed et al^[9]^ demonstrated that children with blood group A are at increased risk for enterotoxigenic E. coli (ETEC) diarrhea. They also proposed that the Lewis blood group “a” antigen (Le^a^) may function as a receptor for ETEC colonization factor antigen I (CFA/I) fimbriae.

Despite progress in understanding the immunohematological role of blood group antigens, their contribution to susceptibility to infectious diseases remains inadequately characterized. There is a pressing need for comprehensive studies to further elucidate these associations. Such insights may enable the development of more personalized medical interventions and enhance our understanding of host-pathogen interactions. Therefore, this study was undertaken to clarify the relationship between blood group phenotypes and susceptibility to sepsis in pediatric patients.

## 2. Materials and methods

This retro-prospective case-control study was conducted at the Children’s Hospital in Taif, Saudi Arabia, between January 2023 and May 2025. Data collection, laboratory analysis, and statistical evaluation were performed during this period. A total of 302 pediatric patients were enrolled, comprising 60 children diagnosed with sepsis (cases) and 242 children with non-septic illnesses (controls). Controls were selected from patients presenting with various conditions unrelated to sepsis and excluded if key clinical or demographic data were missing.

Matching between cases and controls was evaluated statistically using independent t-tests and chi-square tests for age and sex, respectively. No significant differences were observed (*P* >.05), indicating appropriate matching between the groups.

Blood samples were collected from all participants for blood group typing, complete blood count (CBC), microbiological analysis, and antimicrobial susceptibility testing. Patients with single and mixed infections were included in the case group.

For laboratory analysis, samples were obtained in ethylenediaminetetraacetic acid (EDTA) tubes for hematological and blood bank testing, and in pediatric blood culture bottles for microbiological evaluation. The Sysmex XN-1000 Hematology Analyzer (Sysmex Corporation, Kobe, Japan) was used to determine CBC parameters. Blood group typing, including ABO, Rh, and direct agglutination testing, was performed using the ORTHO VISION™ Analyzer (Ortho Clinical Diagnostics, Raritan, NJ, USA), which provides automated and standardized blood group phenotyping.

Blood cultures were processed using the BD BACTEC™ 9120 automated system (Becton Dickinson, Sparks, Sparks Glencoe), which continuously monitors culture bottles for both bacterial and fungal growth, including *Candida* species. Upon positive signaling, bottles were immediately subcultured onto appropriate solid media: blood agar, MacConkey agar, mannitol salt agar, and chocolate agar for bacterial isolation, and Sabouraud dextrose agar for fungal recovery. Bacterial culture plates were incubated aerobically at 37 °C for 18 to 24 hours, whereas chocolate agar plates were incubated under increased CO₂ tension in a candle jar to support the growth of fastidious organisms. Sabouraud dextrose agar plates were incubated at 30 °C for up to 48 hours to facilitate fungal growth and identification. Fungal isolates, primarily *Candida* species, were further identified using the VITEK® 2 Compact System (bioMérieux, France).

Anaerobic cultures were not performed because the study design focused on aerobic bacterial and fungal pathogens typically implicated in pediatric sepsis. The present investigation specifically addressed bloodstream culture–confirmed sepsis; non-blood sources of infection (such as respiratory, urinary, intra-abdominal, or skin/soft-tissue origins) were not evaluated systematically. Demographic, hematological, and microbiological data were retrieved from the hospital’s laboratory information system for analysis and correlation with clinical findings.

Statistical analyses were performed using SPSS version 26 (IBM Corp., Armonk). Chi-square tests, Fisher exact tests, and independent t-tests were used, where appropriate. Statistical significance was set at *P* <.05.

## 3. Results

A total of 302 pediatric patients were included in the study, with a mean age ± standard deviation of 2.59 ± 2.07 years. Participants were categorized according to developmental stage: 22 (7%) were newborns, 118 (39%) were infants, and 162 (54%) were children. Among the 60 patients with sepsis (cases), the mean age was 1.78 ± 1.58 years, with 29 (48.3%) males and 31 (51.7%) females. The control group included 242 children with a mean age of 2.15 ± 2.78 years, comprising 144 males (59.5%) and 98 females (40.5%). No statistically significant differences were observed between cases and controls in terms of age or sex distribution (*P* >.05).

### 3.1. Blood group distribution

In the overall study population, blood group O was the most prevalent, found in 167 participants (55.3%), followed by blood group A in 75 participants (24.8%), blood group B in 47 participants (15.6%), and blood group AB in 13 participants (4.3%). Regarding Rh, 275 participants (91.1%) were Rh-positive and 27 (8.9%) were Rh-negative. Kell antigen typing revealed that 212 participants (70.2%) were Kell-negative, and 90 (29.8%) were Kell-positive.

### 3.2. Complete blood count analysis

**Figure 1.**
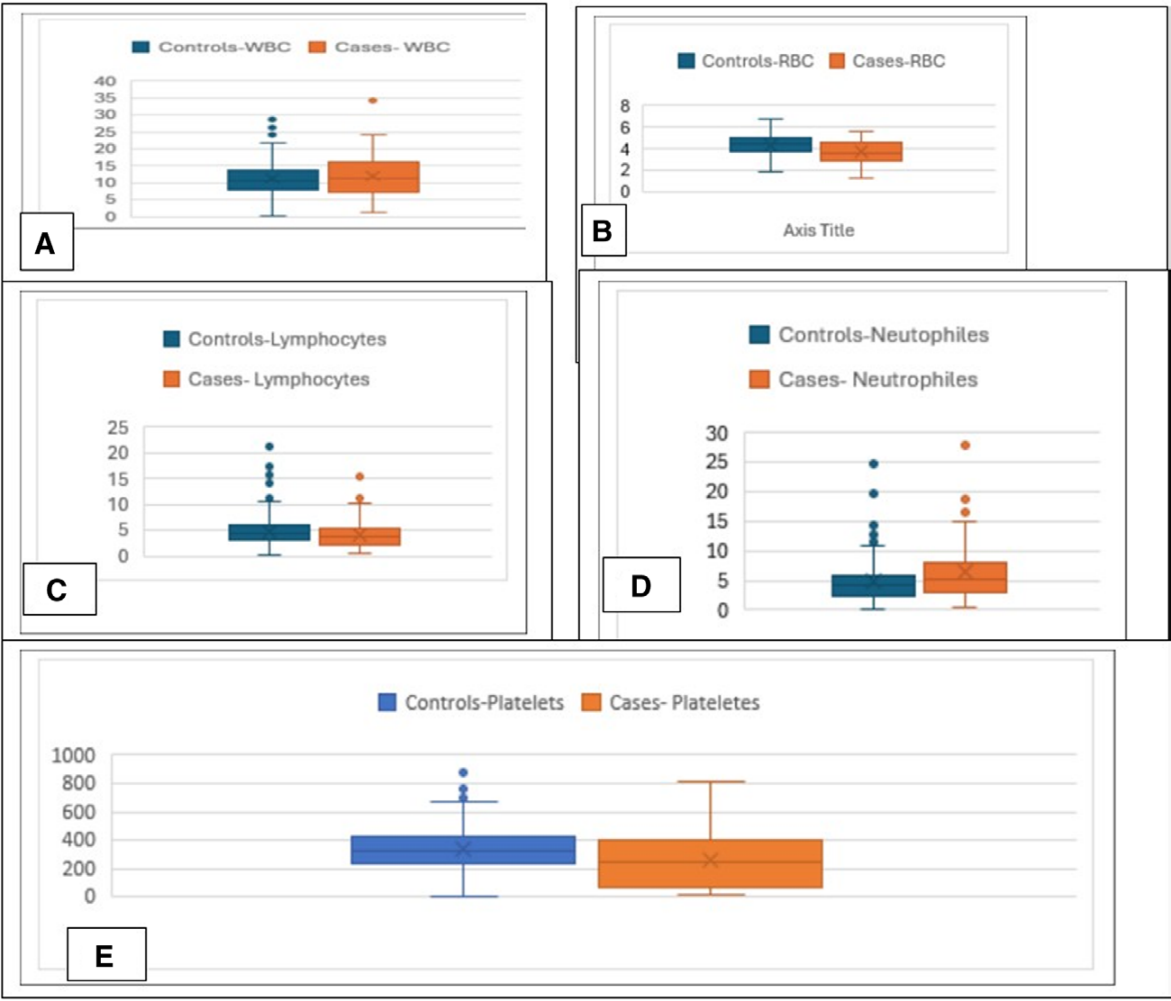
Distribution of complete blood count among study groups.

CBC parameters were analyzed across the different pediatric age groups. A boxplot visualization (Fig. 1) was used to illustrate the distribution of CBC values. The boxes represent the interquartile range (IQR), and the whiskers indicate the full range of values, allowing for comparison of hematological parameters among newborns, infants, and children.

As shown in Figure 1: red blood cells (RBC): Significantly lower in cases (3.73 ± 1.04) compared to controls (4.32 ± 0.92), *P* <.001. WBC (White Blood Cells): Slightly higher in cases (11.96 ± 6.12) vs controls (11.18 ± 4.84), but not statistically significant (*P* = .29). Neutrophils: Significantly higher in cases (6.36 ± 4.91) compared to controls (4.73 ± 3.26), *P* = .002. Lymphocytes: Lower in cases (4.73 ± 2.64) vs controls (5.58 ± 8.83), but not statistically significant (*P* = .19). Platelets: Significantly lower in cases (254.85 ± 188.61) compared to controls (336.02 ± 159.5), *P* = .001.

**Figure 2.**
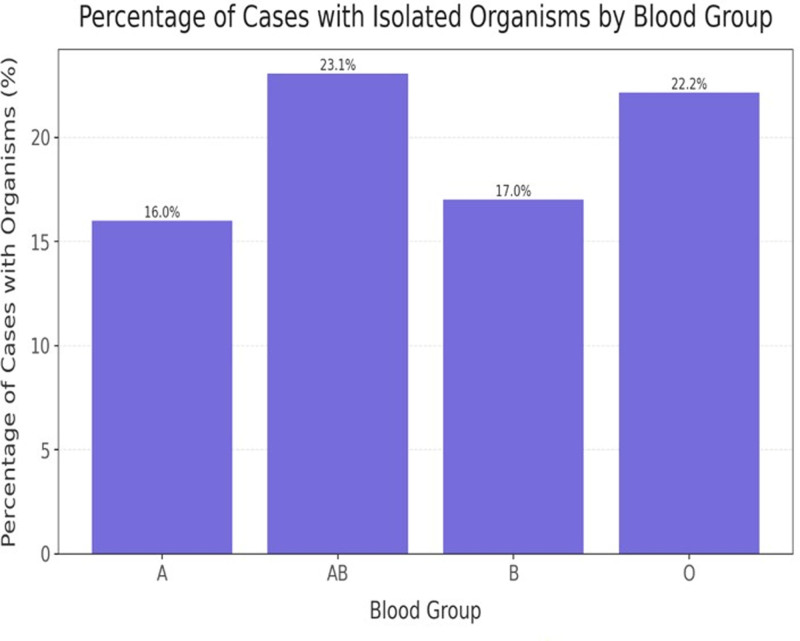
Percentage of cases with isolated microorganisms with different blood groups.

Figure 2 shows percentage of cases with isolated microorganisms with different blood groups

### 3.3. Infection source distribution

**Figure 3.**
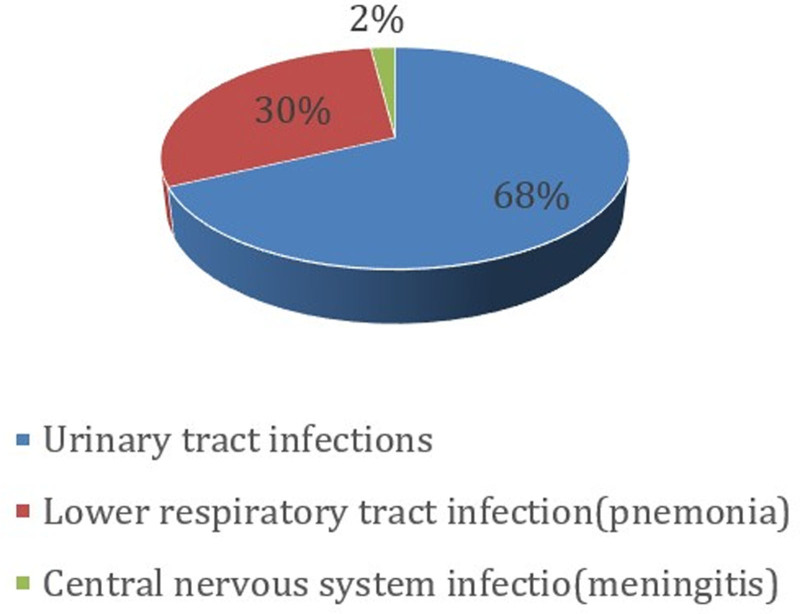
Distribution of infection sources among pediatric sepsis cases.

Figure 3 illustrates the distribution of infection sources among the sepsis cases. Urinary tract infections were the most common, accounting for 41 cases (68%), followed by lower respiratory tract infections (pneumonia) in 18 cases (30%), and central nervous system infection (meningitis) in 1 case (2%).

As shown in Table 1, Gram-positive cocci were the most frequently isolated pathogens among sepsis cases, followed by Gram-negative bacilli, *Candida* species, and mixed infections. A higher proportion of *Candida* species and mixed infections was observed among patients with blood group AB, whereas Gram-positive cocci predominated in Kell-positive individuals. In contrast, Gram-negative bacilli were more common among Rh-positive and Kell-negative patients. Multidrug resistance (MDR) was predominantly observed in gram-negative isolates (70.6%), compared with only 17.6% among Gram-positive cocci (Table 2), indicating a statistically significant association between Gram-negative infections and antimicrobial resistance (*P* <.001). These patterns highlight a potential link between specific blood group phenotypes and pathogen profiles, as well as the higher resistance burden among Gram-negative organisms.

**Table 1:** The association between major and minor blood groups and isolated microorganisms among cases group.

Blood groups	Isolated organisms							
	Gram + ve cocci		Gram - ve Bacilli		Candida spp.		Mixed growth	
	n	Row %	n	Row %	n	Row %	n	Row %
A (n = 12)	6	50.0	5	41.7	1	8.3	0	0.0
B (n = 8)	4	50.0	3	37.5	0	0.0	1	12.5
AB (n = 3)*	0	0.0	0	0.0	2	66.7	1	33.3
O (n = 37)	17	45.9	11	29.7	5	13.5	4	10.8
RH + ve (n = 57)	25	43.9	18	31.6	8	14.0	6	10.5
RH − ve (n = 3)	2	66.7	1	33.3	0	0.0	0	0.0
Kell + ve (n = 17)	11	64.7	3	17.6	1	5.9	2	11.8
Kell—ve (n = 43)	16	37.2	16	37.2	7	16.3	4	9.3

* *P*-value < .01, significant statistical value.

**Table 2:** Association between isolated microorganisms and AST among cases group.

AST	Gram + ve cocci		G -ve bacilli		Mixed growth		*P*-value
	Count	%	Count	%	Count	%	
MDR	3	17.6	12	70.6	2	11.8	.001
Susceptible	2	20	5	50	3	30	
Commensals	22	88	2	8	1	4	

AST = antibiotic sensitivity test, MDR = multidrug resistant.

The association between isolated microorganisms and antimicrobial susceptibility testing among cases group is shown in Table 2.

## 4. Discussion

Our study revealed that blood group O was the most prevalent, accounting for 167 (55.3%) of participants, followed by blood group A with 75 (24.8%), blood group B with 47 (15.6%), and blood group AB with 13 (4.3%). This blood group distribution is consistent with a study conducted in the Makkah region, which reported blood group O+ as the most prevalent at 43.8%, followed by blood group A+ at 26.28% and blood group B+ at 17.66%.^[10]^ Similarly, our results align with those of a study in the southern region of Saudi Arabia, where blood group O was most common at 56.8%, followed by A at 33.4%, B at 6%, and AB at 3.8%.^[11]^ The consistency across these regions may reflect shared genetic backgrounds, limited migration, and population stability.

Regarding Rh factor, 275 (91.1%) of our participants were Rh-positive, while 27 (8.9%) were Rh-negative, aligning with previous findings in Makkah (92.2% Rh-positive)^[10]^ and the southwestern region (93% Rh-positive),^[11]^ reinforcing the high prevalence of Rh positivity in Saudi Arabia. As for the Kell phenotype, 212 (70.2%) were Kell-negative and 90 (29.8%) were Kell-positive. This is comparable to findings by Khojah et al in Makkah,^[12]^ where K − k + accounted for 46.7%, and Felimban et al in Jeddah,^[13]^ where 83.7% were K − k+. Similarly, Alalshaikh et al in Riyadh^[14]^ found 81.5% Kell-negative and only 3% Kell-positive individuals. These variations may be partially attributed to differing sample sizes and regional genetic diversity.

Regarding hematologic parameters, Figure 1 demonstrates that RBC counts were significantly lower in sepsis cases (3.73 ± 1.04) than in controls (4.32 ± 0.92), with *P* <.001. This may be attributed to infection-related hemolysis, bone marrow suppression, or inflammatory cytokine–induced erythropoietin resistance, all of which are common in sepsis-related anemia.^[15]^ Neutrophil counts were significantly elevated in the sepsis group (6.36 ± 4.91) versus the control group (4.73 ± 3.26), with *P* = .002. This aligns with findings in children with invasive meningococcal infections, where 63% had absolute neutrophil counts ≥ 10,000/mm³,^[16]^ and in neonates with sepsis, where 9 out of 11 had elevated neutrophils.^[17]^ Such neutrophilia reflects activation of the innate immune response, an early marker of bacterial infection severity.

Platelet counts were also significantly lower in sepsis cases (254.85 ± 188.61) than in controls (336.02 ± 159.5), with *P* = .001. Thrombocytopenia in sepsis is known to predict poor prognosis, as supported by Sherkatolabbasieh et al,^[18]^ who showed a correlation between platelet and CRP levels. In agreement, Zhang et al^[19]^ found that 34.4% of pediatric septic shock patients developed thrombocytopenia, which was associated with increased mortality and organ failure. This reduction in platelets may result from platelet consumption, bone marrow suppression, or immune-mediated destruction during systemic inflammation.

In Figure 2, the AB blood group showed the highest percentage of cases with isolated organisms (23.08%), followed by O (22.16%), B (17.02%), and A (16%). However, due to the small sample size for AB (n = 13), these results lack statistical significance (*P* >.05). This is in agreement with Acun et al,^[20]^ who reported no correlation between ABO blood group and sepsis. Nevertheless, Table 1 indicated a significant association between AB blood group and candidemia, corroborated by Cakir et al,^[21]^ though contradicted by Thanomsridetcha et al.^[22]^ This may be due to the absence of anti-A and anti-B antibodies in AB individuals, potentially compromising innate immunity and allowing easier fungal adhesion or colonization, particularly by *Candida* species.

In terms of microbial profiles, Gram-positive cocci accounted for the highest proportion of isolates (45%), followed by Gram-negative bacilli (31.6%), *Candida* species (13.3%), and mixed infections (10%). This partially aligns with Alharbi,^[23]^ who reported a higher Gram-positive rate (67.5%) at King Abdulaziz University Hospital. Banawas et al^[24]^ reported 55.9% Gram-positive and 44.1% Gram-negative bacteria in Riyadh, while Alarjani et al^[25]^ found 64.22% Gram-positive and 32.5% Gram-negative isolates. Conversely, Al Thaqafi et al^[26]^ reported a higher incidence of candidemia, with *C. albicans* representing 34.1% and non-albicans species 65.9%. The predominance of Gram-positive cocci in our study may reflect their frequent involvement in catheter-associated and community-acquired pediatric sepsis, while the notable proportion of *Candida* species suggests the growing role of fungal infections in hospitalized or antibiotic-exposed children.

Antibiotic susceptibility testing (Table 2) revealed a significant association between Gram-negative organisms and multidrug resistance (MDR), with 12 (70.6%) of Gram-negative isolates exhibiting MDR, compared to only 3 (16.7%) of Gram-positive isolates. Saeedi et al^[27]^ similarly reported a 42% MDR rate in pediatric patients, with even higher rates in NICU/PICU settings. Almutairi et al^[28]^ found that 45.4% of *S. aureus* isolates were methicillin-resistant (MRSA), while Alabdullatif^[29]^ reported 16.44% MDR and 24.07% MRSA prevalence in Gram-positive isolates from central Saudi Arabia. The high MDR rates observed, particularly among Gram-negative bacilli, are likely due to widespread antibiotic use, horizontal gene transfer, and selective pressure within hospital environments. These findings reinforce the need for ongoing antimicrobial stewardship and surveillance in pediatric care.

The most common source of septicemia was urinary tract infection, in agreement with the study done by Hsiang-Chin et al.^[30]^ This may be explained by the higher susceptibility of young children to ascending urinary tract infections due to immature immune defenses and anatomical factors, especially in females.

Limitations: This single-center study with a modest sample size may limit the generalizability of its findings. It focused solely on bloodstream culture–confirmed sepsis; data on other infection sources (e.g., urinary, respiratory, or soft-tissue) were not collected. Including more neonates and applying advanced microbial identification methods in future multicenter studies could enhance understanding of microbial patterns and validate these associations across Saudi Arabia.

A larger and more diverse sample size across multiple regions of Saudi Arabia is recommended to validate the associations identified in this study. As this investigation was limited to bloodstream infections, data on other potential infection sources (e.g., urinary, respiratory, abdominal, or skin/soft-tissue) were not collected, and the role of alternative sepsis origins could not be evaluated. Future studies should also aim to include a greater number of neonates and apply advanced microbial identification techniques to gain deeper insights into pathogen profiles and their clinical implications. Moreover, prospective multicenter research that integrates both laboratory and clinical parameters would help clarify how blood group phenotypes relate not only to susceptibility but also to the clinical presentation and severity of pediatric sepsis across diverse populations

## 5. Conclusions

Blood group O was the most prevalent and the least was AB, also Rh-positive and Kell-negative were more prevalent. AB group had statistically significant association with Candida species and mixed infections; Kell-positive individuals showed more Gram-positive cocci isolation. Multidrug-resistant organisms were mainly among Gram-negative bacilli. Most commensals were Gram-positive cocci.

## Acknowledgments

The authors thank the technicians of Children hospital in Taif for their help during performing the practical part of the study and also thank Taif University Project No. (TU-DSPP-2024-81).

## Author contributions

**Conceptualization:** Howaida M. Hagag, Maha M. Bakhuraysah, Abdulrahman A. Almalki, Ahmad M. Homsi, Maram Jameel Hulbah, Khadiga A. Ismail, Usama Mahmoud Marzouk, Abdalla A. Elnour, Mahmoud Yehyia Sleem, Abdulkarim Hasan.

**Data curation:** Howaida M. Hagag, Maha M. Bakhuraysah, Mustafa A. Alswat, Abdulrahman A. Almalki, Ahmad M. Homsi, Narjes A. Alhassan, Shmokh K. Altalhi, Amjad S. Alrubaie, Maram Jameel Hulbah, Khadiga A. Ismail, Ali A. Lafi Alghamdi, Abdulkarim Hasan.

**Formal analysis:** Howaida M. Hagag, Mustafa A. Alswat, Ahmad M. Homsi, Mahmoud Yehyia Sleem, Raed A. Alharbi, Ali A. Lafi Alghamdi, Abdulkarim Hasan.

**Funding acquisition:** Khadiga A. Ismail.

**Investigation:** Howaida M. Hagag, Narjes A. Alhassan, Shmokh K. Altalhi, Nada A. Albogami, Maram Jameel Hulbah, Reem Amr Ramadan, Khadiga A. Ismail, Abdalla A. Elnour, Mahmoud Yehyia Sleem, Raed A. Alharbi, Ali A. Lafi Alghamdi, Salem A. Turki Alghamdi, Abdulkarim Hasan.

**Methodology:** Howaida M. Hagag, Mustafa A. Alswat, Narjes A. Alhassan, Shmokh K. Altalhi, Nada A. Albogami, Maram Jameel Hulbah, Khadiga A. Ismail, Usama Mahmoud Marzouk, Abdalla A. Elnour, Mahmoud Yehyia Sleem, Raed A. Alharbi, Salem A. Turki Alghamdi, Abdulkarim Hasan.

**Project administration:** Howaida M. Hagag.

**Resources:** Maha M. Bakhuraysah, Abdulrahman A. Almalki, Ahmad M. Homsi, Shmokh K. Altalhi, Amjad S. Alrubaie, Reem Amr Ramadan, Salem A. Turki Alghamdi.

**Software:** Maha M. Bakhuraysah, Reem Amr Ramadan, Usama Mahmoud Marzouk, Raed A. Alharbi.

**Visualization:** Maha M. Bakhuraysah, Mustafa A. Alswat, Narjes A. Alhassan, Nada A. Albogami, Amjad S. Alrubaie, Ali A. Lafi Alghamdi.

**Writing – original draft:** Howaida M. Hagag, Maha M. Bakhuraysah, Abdulrahman A. Almalki, Narjes A. Alhassan, Shmokh K. Altalhi, Nada A. Albogami, Amjad S. Alrubaie, Khadiga A. Ismail.

**Writing – review & editing:** Howaida M. Hagag, Mustafa A. Alswat, Abdulrahman A. Almalki, Ahmad M. Homsi, Nada A. Albogami, Amjad S. Alrubaie, Maram Jameel Hulbah, Reem Amr Ramadan, Khadiga A. Ismail, Usama Mahmoud Marzouk, Abdalla A. Elnour, Mahmoud Yehyia Sleem, Raed A. Alharbi, Ali A. Lafi Alghamdi, Salem A. Turki Alghamdi.
